# Supplementary material for: Characteristics, Outcomes, and Factors Affecting Mortality in Hospitalized Patients with CAP Due to Different Variants of SARS-CoV-2 and Non-COVID-19 CAP
Source: J Clin Med. 2023 Feb 9;12(4):1388. doi: 10.3390/jcm12041388 (PMC9964315; doi:10.3390/jcm12041388)
Supplement: Supplementary file 1 [file jcm-12-01388-s001.zip › jcm-2188299-supplementary.pdf]

Supplementary materials

The definition of pneumonia case according to ATS/IDSA guideline

1. hospital-acquired pneumonia (HAP) is defined as pneumonia that occurs 48 hours or more after admission, which was not incubating at the time of admission
2. ventilator–associated pneumonia (VAP) refers to pneumonia that arises more than 48–72 hours after endotracheal intubation
3. healthcare-associated pneumonia (HCAP) includes any patient who was hospitalized in an acute care hospital for two or more days within 90 days of the infection; resided in a nursing home or long-term care facility; received recent intravenous antibiotic therapy, chemotherapy, or wound care within the past 30 days of the current infection; or attended a hospital or hemodialysis clinic
4. community-acquired pneumonia (CAP) defined as any other pneumonia not captured by the above categories

Pneumonia definition

Criteria was modified from CDC/NHSN surveillance definition of health care–associated infection. For clinical and radiological diagnosis of pneumonia must fulfill both radiology and clinical criteria

- **Radiology**
  - **Patients without underlying: One or more** chest radiographs with at least 1 of the following,
    - New or progressive and persistent infiltrate
    - Consolidation
    - Cavitation
  - **Patient with underlying pulmonary or cardiac disease** (eg, respiratory distress syndrome, bronchopulmonary dysplasia, pulmonary edema, or chronic obstructive pulmonary disease), **One baseline chest radiograph with one or more serial chest radiograph** with at least 1 of the following
    - New or progressive and persistent infiltrate
    - Consolidation
    - Cavitation
- **Clinical Sign/Symptom**
  - **FOR ANY PATIENT, at least 1 of the following:**
    - Fever (>38°C or >100.4°F) with no other recognized cause
    - Leukopenia (<4000 WBC/mm^3) or leukocytosis (≥12,000 WBC/mm^3)
    - For adults >70 years old, altered mental status with no other recognized cause
  - **AND** at least 2 of the following:
    - New onset of purulent sputum or change in character of sputum or increased respiratory secretions or increased suctioning requirements
    - New onset or worsening cough, or dyspnea, or tachypnea
    - Rales or bronchial breath sounds
    - Worsening gas exchange (eg, O_2 desaturations [eg, PaO2/FiO2 ≤ 240], increased oxygen requirements, or increased ventilator demand)
